# Physics of Composites for Low-Frequency Magnetoelectric Devices

**DOI:** 10.3390/s22134818

**Published:** 2022-06-25

**Authors:** Mirza Bichurin, Oleg Sokolov, Sergey Ivanov, Viktor Leontiev, Dmitriy Petrov, Gennady Semenov, Vyacheslav Lobekin

**Affiliations:** Yaroslav-the-Wise Novgorod State University, 173003 Veliky Novgorod, Russia; oleg.sokolov@novsu.ru (O.S.); s243962@std.novsu.ru (S.I.); viktor.leontev@novsu.ru (V.L.); petrovd@okbplaneta.ru (D.P.); gennady.semenov@novsu.ru (G.S.); slavalobekin@gmail.com (V.L.)

**Keywords:** magnetoelectric effect, magnetoelectric composite, magnetoelectric voltage coefficient, electromechanical resonance, resonance mode

## Abstract

The article discusses the physical foundations of the application of the linear magnetoelectric (ME) effect in composites for devices in the low-frequency range, including the electromechanical resonance (EMR) region. The main theoretical expressions for the ME voltage coefficients in the case of a symmetric and asymmetric composite structure in the quasi-static and resonant modes are given. The area of EMR considered here includes longitudinal, bending, longitudinal shear, and torsional modes. Explanations are given for finding the main resonant frequencies of the modes under study. Comparison of theory and experimental results for some composites is given.

## 1. Introduction

At present, magnetoelectric (ME) composites are extensively studied [1,2,3]. Researchers pay main attention to layered composites because of the possibility of obtaining the maximum ME effect on their basis. Because of the content of magnetic and electrical (piezoelectric or ferroelectric) components in its structure, the ME composite is a multifunctional material and considerably interests developers of ME devices compared to conventional magnetic and electrical materials. The presence of a magnetic component makes it possible to change the magnetic properties of the composite by applying an external electric field, and the electrical properties change when exposed to an external magnetic field. Depending on the external applied fields in ME composites, the direct and inverse ME effects are distinguished. In the case of the direct effect, electric polarization is induced in the composite when it is exposed to a magnetic field, whereas, in the case of an inverse effect, magnetization occurs when exposed to an electric field. The main characteristic of the ME composite in the case of a direct effect is the ME voltage coefficient, which is the ratio of the induced electric field to the alternating magnetic field acting on the composite. There are numerous works devoted to the calculation of individual characteristics of ME composites [4,5,6,7,8,9,10,11,12,13,14,15,16,17,18,19,20] and examples of the development of various ME devices: sensors [21,22,23,24,25,26,27,28], gyrators [29,30], harvesters [31,32,33], antennas [34,35], and microwave devices [3,36]. The information in the literature concerns the calculations of the ME effect for individual electromechanical (EMR) regimes [6,7,8,9,10,11,12,13,14,15,16,17,18,19], then further in the article we will carry the comparison of the obtained theoretical results with these data out. As an example of a developed perspective ME device, we can discuss the ME magnetic field sensor [22]. The great interest in this device is because, having a simple three-layer Metglas-PMN-PT-Metglas structure, in the near future it can replace a complex electronic device such as a SQUID operating at helium temperature, since comparable values for giant ME voltage coefficient of 4.26 × 10^4^ V cm^−1^ Oe^−1^ and the equivalent magnetic noise of 2.89 fT Hz^−1/2^ at EMR frequency on this structure have already been achieved. If we consider its small weight and size parameters and operation at room temperature, then we can expect widespread use of the ME magnetic field sensor in areas such as biomedicine [37,38,39]. Next, we should briefly emphasize the main characteristics of other new ME devices, in which they can compete with well-known serial devices. For ME gyrators, which are developed for the purpose of possible replacement of current and voltage transformers, the efficiency of field conversion at the level of 90% has already been obtained [30]. ME harvesters, unlike mechanical/vibrating and piezoelectric counterparts, allow using all types of energy in the collection: mechanical, piezoelectric, magnetic, and electromagnetic, including microwave. So far, we have received of 18,700 mW/m^3^ by the join application of an AC magnetic field and mechanical vibrations [33]. Wide-range ME antennas, operating on the principle of acoustoelectronic conversion, can significantly reduce the weight and size parameters and radiation power, which is very important for their possible use in underground and underwater communications [34]. New ME control devices in the microwave range, such as filters, attenuator-isolators, and phase shifters, unlike ferrite analogs, have dual control of their parameters by the magnetic and electric fields, which makes it possible to increase their speed and manufacturability [3]. 

As already noted, there are works devoted to the calculation of individual EMR regimes of the low-frequency ME effect. The authors of the article believe that it is useful for developers of ME devices to have the results of calculations for all modes in one place for more efficient operation. Therefore, the purpose of this work is to consider from a unified standpoint the theory of the direct ME effect in composites in the low-frequency range, including all modes of EMR resonance: longitudinal, bending, longitudinal-shear, and torsional ones. In order to compare the calculated and experimental results, data are presented for symmetric/three-layer and asymmetric/two-layer structures based on Metglas and various piezoelectrics: PZT, lithium niobate, and gallium arsenide. The obtained relations may be of interest in the analysis of the properties of the ME composite and in choosing it as the basis of the developed ME device.

The structure of the article is as follows. The purpose of the article is indicated in the introduction. Section 2 is devoted to the consideration of the longitudinal and bending modes in symmetric and asymmetric ME structures. Section 3 describes the longitudinal shear and torsional modes in such structures. Note that in Section 2 and Section 3, as a special case, the quasi-static regime is also considered. A discussion of the accuracy of the formulas for the resonant frequencies of various EMR modes is included in Section 4. Section 5 discusses the conclusions of the article.

## 2. Longitudinal and Bending Modes

### 2.1. Symmetric ME Structure

In a symmetric ME structure, excitation of the bending mode of the ME effect is impossible. Therefore, we first consider the general case of a longitudinal mode for an arbitrary frequency of an alternating magnetic field, which also includes the resonant mode, and the expression for the ME voltage coefficient for the quasi-static case is obtained from the general expression using the passage to the limit, letting the frequency tend to zero.

#### 2.1.1. Resonance Mode

**Figure 1:**
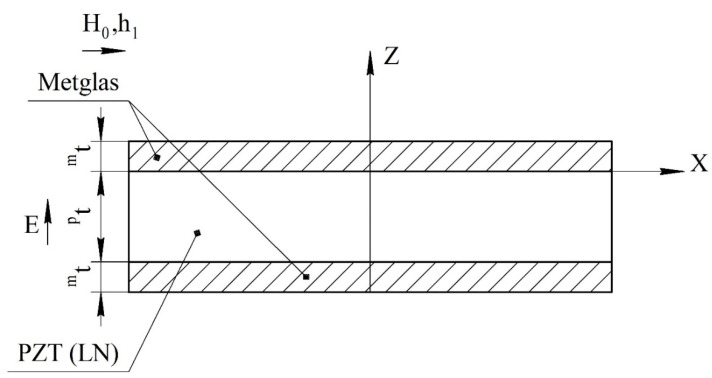
Symmetric tri-layer magnetoelectric composite for the calculation of longitudinal mode. Magnetic fields are parallel to each other and lie in the plane of structure, the electric field is perpendicular to the plane of structure, and *^p^t* and *^m^t* are the thicknesses of piezoelectric and magnetostrictive layers.

We consider a magnetoelectric composite in the form of a thin narrow plate. Layers of the magnetostrictive phase of the same thickness are located above and below the piezoelectric layer. The ME structure created in this way is symmetrical. The *X* axis is directed along the length of the plate, and the *Z* axis is perpendicular to the sample plane (Figure 1).

We consider small mechanical oscillations in a composite under the influence of a small external variable magnetic field. In the presence of a constant magnetic field, the strengths of both fields are directed along the *l (x)* axis:

(1) $$h_{1}\left(t\right)=h_{1}e^{i\omega t},$$

where *h*_1_ (*t*) is an external variable magnetic field, and ω is a cyclic frequency of the external alternating magnetic field.

The material Equation for the piezoelectric layer is given by:

(2) $${}^{p}S_{1}=d_{31}E_{3}+{}^{p}S_{11}{}^{p}T_{1},$$

where *^p^S*_1_ is the strain tensor component of piezoelectric phase; *d*_31_ is piezoelectric coefficient; *E*_3_ is component of the vector of the electric field; *^p^s*_11_ is compliance tensor component of the piezoelectric phase; *^p^T*_1_ is the stress tensor component of the piezoelectric phase.

The longitudinal component of the stress tensor in a piezoelectric phase can be expressed as:

(3) $${}^{p}T_{1}=\frac{1}{{}^{p}S_{11}}{}^{p}S_{1}-\frac{d_{31}}{{}^{p}S_{11}}E_{3}.$$

The longitudinal component of the stress tensor of the magnetostrictive phase is given by:

(4) $${}^{m}T_{1}={}^{m}Y\left({}^{m}S_{1}-q_{11}{\tilde{h}}_{1}\right),$$

where *^m^T*_1_ is the stress tensor component of the magnetostrictive phase; *^m^Y* is their Young’s module; *^m^S*_1_ is the strain tensor component of magnetostrictive layer; *q*_11_ is piezomagnetic coefficient; ${\tilde{h}}_{1}$ is the intensity of the alternating magnetic field inside the ferromagnet.

The constitutive Equations for the ferromagnetic phase are given by:

(5) $$B_{1}=\mu \mu_{0}{\tilde{h}}_{1}+q_{11}{}^{m}T_{1}$$

(6) $$\mu \mu_{0}h_{1}=\mu \mu_{0}{\tilde{h}}_{1}+q_{11}{}^{m}T_{1},$$

where *B_1_* is magnetic induction; *μ* is magnetic permeability of an isotropic medium; *μ*_0_ is magnetic constant; *h*_1_ is the intensity of an external alternating magnetic field away from the ferromagnet.

Express ${\tilde{h}}_{1}$ from Equation (6):

(7) $${\tilde{h}}_{1}=h_{1}-\frac{q_{11}}{\mu \mu_{0}}{}^{m}T_{1}$$

Substituting Equation (7) in Equation (4), we get:

(8) $${}^{m}T_{1}={}^{m}Y\left({}^{m}S_{1}-q_{11}\left[h_{1}-\frac{q_{11}}{\mu \mu_{0}}{}^{m}T_{1}\right]\right)$$

Express *^m^T*_1_ from Equation (8):

(9) $${}^{m}T_{1}={}^{m}Y^{B}{}^{m}S_{1}-{\bar{q}}_{11}h_{1},$$

where:

(10) $$\begin{array}{l} {}^{m}Y^{B}=\frac{{}^{m}Y}{1-{}^{m}K_{11}^{2}}\\ {\bar{q}}_{11}={}^{m}Y^{B}q_{11} \end{array}$$

where *^m^Y^B^* is the Young’s modulus under constant magnetic induction; *^m^K*_11_ is the coefficient of magnetomechanical coupling.

The square of the coefficient of magnetomechanical coupling is:

(11) $${}^{m}K_{11}^{2}=\frac{{}^{m}Yq_{11}^{2}}{\mu \mu_{0}}$$

Since the length of the composite is much greater than its width and height, longitudinal vibrations arise in it.

In accordance with the condition of the problem:

(12) $${}^{m}S_{1}={}^{p}S_{1}=S_{1}$$

The longitudinal component of the composite stress tensor is:

(13) $$T_{1}={}^{m}\nu {}^{m}T_{1}+{}^{p}\nu {}^{p}T_{1}=c_{11}S_{1}-{}^{m}\nu {\bar{q}}_{11}h_{1}-{}^{p}\nu \frac{d_{31}}{{}^{p}S_{11}}E_{3},$$

where volume fractions of the *^p^ν* piezoelectric and *^m^ν* magnetostrictive phases are:

(14) $$\begin{array}{l} {}^{p}\nu =\frac{{}^{p}t}{{}^{p}t+2{}^{m}t}\\ {}^{m}\nu =\frac{2{}^{m}t}{{}^{p}t+2{}^{m}t} \end{array}$$

where *^p^t* and *^m^t* are the thicknesses of piezoelectric and magnetostrictive layers and effective composite stiffness coefficient:

(15) $$c_{11}=\frac{{}^{p}\nu }{{}^{p}S_{11}}+{}^{m}\nu {}^{m}Y^{B}$$

Composite effective density can be obtained from:

(16) $$\rho ={}^{p}\nu^{p}\rho +{}^{m}\nu^{m}\rho ,$$

where *^p^ρ*, *^m^ρ* are density of the piezoelectric and magnetostrictive phases, respectively.

The longitudinal component of strain tensor is:

(17) $$S_{1}=\frac{\partial U_{x}}{\partial x},$$

where *Ux* is longitudinal component of the strain vector.

Consider the Equation of motion for deformations:

(18) $$\rho \frac{\partial^{2}U_{x}}{\partial t^{2}}=\frac{\partial T_{1}}{\partial x}$$

Substituting Equation (13) in Equation (18), we get:

(19) $$-\rho \omega^{2}U_{x}=c_{11}\frac{\partial^{2}U_{x}}{\partial x^{2}}$$

The solution of this Equation is obtained as:

(20) $$U_{x}=A\cos\left(kx\right)+B\sin\left(kx\right),$$

where the wave number is:

(21) $$k=\sqrt{\frac{\rho }{c_{11}}}\omega ,$$

*A*, *B* are unknown constants.

Then:

(22) $$S_{1}=\frac{\partial U_{x}}{\partial x}=\left(B\cos\left(kx\right)-A\sin\left(kx\right)\right)k$$

(23) $$T_{1}=c_{11}S_{1}-{}^{m}\nu {\bar{q}}_{11}h_{1}-{}^{p}\nu \frac{d_{31}}{{}^{p}S_{11}}E_{3}=c_{11}\left(B\cos\left(kx\right)-A\sin\left(kx\right)\right)k-{}^{m}\nu {\bar{q}}_{11}h_{1}-{}^{p}\nu \frac{d_{31}}{{}^{p}S_{11}}E_{3}$$

To obtain the constant *A* and *B,* we use the equilibrium conditions for a free sample:

(24) $$\begin{array}{l} {T_{1}|}_{x=-\frac{l}{2}}=0\\ {T_{1}|}_{x=\frac{l}{2}}=0 \end{array}$$

where *l* is length of the ME structure.

Substituting Equation (23) in Equation (24):

(25) $$\begin{array}{l} c_{11}\left(B\cos\left(\eta \right)+A\sin\left(\eta \right)\right)k-{}^{m}\nu {\bar{q}}_{11}h_{1}-{}^{p}\nu \frac{d_{31}}{{}^{p}S_{11}}E_{3}=0\\ c_{11}\left(B\cos\left(\eta \right)-A\sin\left(\eta \right)\right)k-{}^{m}\nu {\bar{q}}_{11}h_{1}-{}^{p}\nu \frac{d_{31}}{{}^{p}S_{11}}E_{3}=0 \end{array}$$

where:

(26) $$\eta =\frac{kl}{2}$$

we get:

(27) $$\begin{array}{l} A=0\\ B=\frac{{}^{m}\nu {\bar{q}}_{11}{}^{p}S_{11}h_{1}+{}^{p}\nu d_{31}E_{3}}{{}^{p}S_{11}c_{11}k\cos\left(\eta \right)} \end{array}$$

The transverse component of the electric displacement vector can be obtained from:

(28) $$D_{3}=\varepsilon \varepsilon_{0}E_{3}+d_{31}{}^{p}T_{1}=\varepsilon \varepsilon_{0}E_{3}+d_{31}\left[\frac{1}{{}^{p}S_{11}}S_{1}-\frac{d_{31}}{{}^{p}S_{11}}E_{3}\right]=\left[\varepsilon \varepsilon_{0}-\frac{d_{31}^{2}}{{}^{p}S_{11}}\right]E_{3}+\frac{d_{31}}{{}^{p}S_{11}}S_{1},$$

where ε is dielectric permittivity of the medium; ε_0_ is electrical constant.

The transverse component of the electric field strength vector can be found from the condition that the electric induction flux through the interface between the upper layer of the magnetostrictive phase and the piezoelectric is equal to zero:

(29) $$\int_{-\frac{l}{2}}^{\frac{l}{2}}D_{3}dx=0.$$

Substituting Equation (28) in Equation (29):

(30) $$\left[\varepsilon \varepsilon_{0}-\frac{d_{31}^{2}}{{}^{p}S_{11}}\right]E_{3}l+\frac{2d_{31}}{{}^{p}S_{11}}B\sin\left(\eta \right)=0$$

and substituting Equation (27) in Equation (30):

(31) $$\left[\varepsilon \varepsilon_{0}-\frac{d_{31}^{2}}{{}^{p}S_{11}}\right]E_{3}l+\frac{2d_{31}}{{}^{p}S_{11}}\sin\left(\eta \right)\frac{{}^{m}\nu {\bar{q}}_{11}{}^{p}S_{11}h_{1}+{}^{p}\nu d_{31}E_{3}}{{}^{p}S_{11}c_{11}k\cos\left(\eta \right)}=0$$

from Equation (31), *E_3_* is obtained as:

(32) E3=−νmq¯11d31pS11tan(η)εε0pS112c11η+d312[pνtan(η)−c11pS11η]h1

As the electric field exists only in the piezoelectric phase, the voltage is given by the following equation:

(33) $$U=E_{3}{}^{p}t$$

Average electric field strength in ME composite is:

(34) $$\bar{E}=\frac{U}{2{}^{m}t+{}^{p}t}=\frac{E_{3}{}^{p}t}{2{}^{m}t+{}^{p}t}={}^{p}\nu E_{3}$$

Then, the ME voltage coefficient is obtained as:

(35) $$\alpha_{E}=\frac{\bar{E}}{h_{1}}=-\frac{{}^{m}\nu {}^{p}\nu {\bar{q}}_{11}d_{31}{}^{p}S_{11}\tan\left(\eta \right)}{\varepsilon \varepsilon_{0}{}^{p}S_{11}^{2}c_{11}\eta +d_{31}^{2}\left[{}^{p}\nu \tan\left(\eta \right)-c_{11}{}^{p}S_{11}\eta \right]}$$

**Figure 2:**
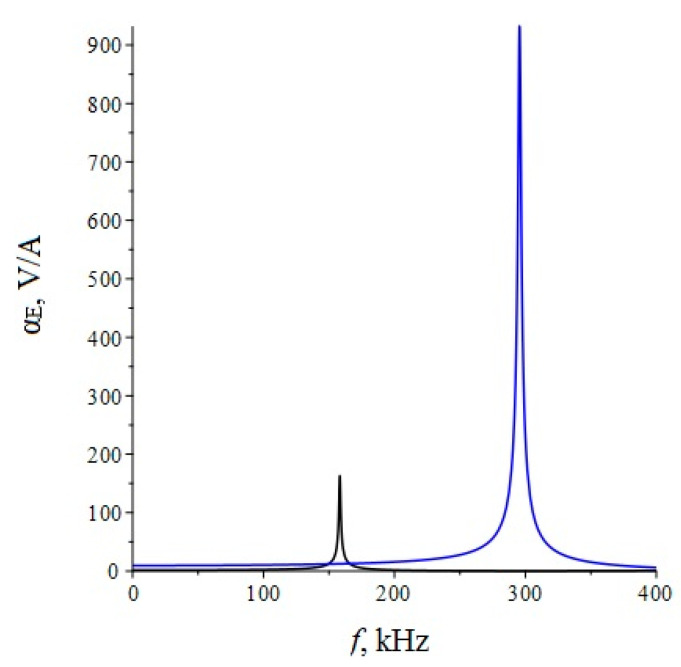
Theoretical dependence of the ME voltage coefficient on the frequency of the alternating magnetic field. Black color of the line is PZT, blue is LN cut y + 128°.

Below, Figure 2 shows the dependence of the ME voltage coefficient on the frequency of the alternating magnetic field for two cases, when PZT and a cut of lithium niobate y + 128° [13,19] are taken as the piezoelectric phase. Metglas is taken as the magnetostrictive phase. For the calculation, the following thicknesses of Metglas *^m^t* = 29 µm and piezoelectric *^p^t* = 0.5 mm are taken, and the length of ME composite is l = 10 mm. To take into account losses in the calculation, it is assumed: *ω* = 2*π*(1 + (1/2*Q*)*i*)*f*, where *Q* is the quality factor of the resonant system. For this calculation, the value of the quality factor *Q* = 130 was taken.

The fundamental resonant frequency for this case is:

(36) $$f_{r}=\frac{1}{2l}\sqrt{\frac{c_{11}}{\rho }}$$

In [7,8], the corresponding theory for the longitudinal mode of the ME effect in the EMR region was used, and it showed its good agreement with the experiment.

#### 2.1.2. Quasi-Static Mode

Assuming in Equation (35) the frequency f is equal to zero, we obtain:

(37) $$\alpha_{E}=-\frac{{}^{m}\nu {}^{p}\nu {\bar{q}}_{11}d_{31}{}^{p}S_{11}}{\varepsilon \varepsilon_{0}{}^{p}S_{11}^{2}c_{11}+d_{31}^{2}\left[{}^{p}\nu -c_{11}{}^{p}S_{11}\right]}=\frac{{}^{m}\nu {}^{p}\nu {\bar{q}}_{11}d_{31}}{{}^{m}\nu {}^{m}Y^{B}d_{31}^{2}-\varepsilon \varepsilon_{0}{}^{p}S_{11}c_{11}}$$

**Figure 3:**
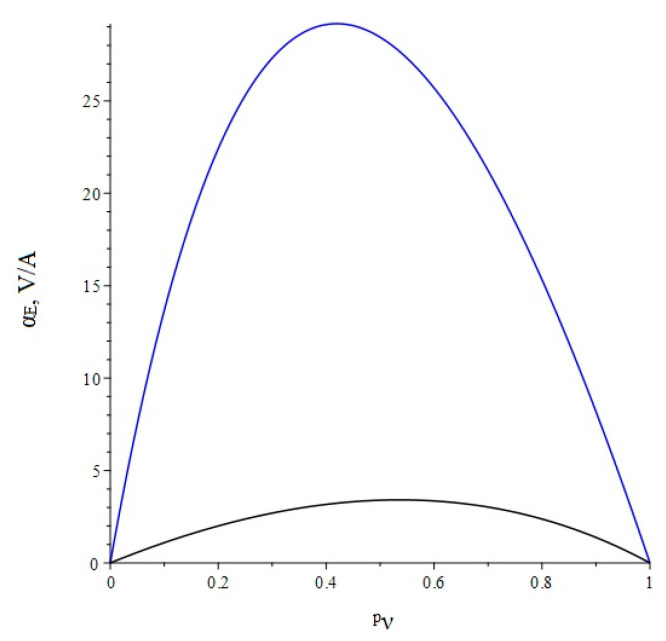
Theoretical dependence of the ME voltage coefficient on the volume fraction of the piezoelectric. Black color of the line is PZT, blue is LN cut y + 128°.

Below, Figure 3 shows the dependence of the ME voltage coefficient on the volume fraction of the piezoelectric for two cases, when PZT and a cut of lithium niobate y + 128° are taken as the piezoelectric phase. Metglas is taken as the magnetostrictive phase.

In [9,10], the corresponding theory for the longitudinal mode of the ME effect in the quasi-static regime was applied, and it showed good agreement with the experiment.

### 2.2. Asymmetric ME Structure

#### 2.2.1. Resonance Regime of the Longitudinal Mode

**Figure 4:**
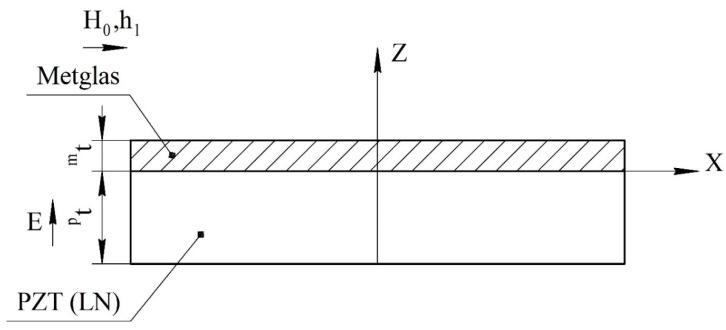
Asymmetrical two-layer magnetoelectric composite for calculation of longitudinal and bending modes. All designations are the same as in Figure 1.

For an asymmetric ME structure in the resonant mode of the longitudinal ME mode, the voltage coefficient can be found from Equation (35), and only in Equation (14) is it necessary to remove the number 2 before *^m^t*. The fundamental resonant frequency for this case can be found in Equation (36). The ME structure shown at Figure 4.

#### 2.2.2. Resonant Mode of the Bending Mode

We consider bending oscillations in a two-layer magnetostrictive-piezoelectric structure. We assume that the sample has the form of a thin bar, whose thickness and width are much less than the length. In this case, we can consider only one component of the stress and strain tensor. 

The full thickness of the composite:


(38) $$t={}^{p}t+{}^{m}t$$

The volume fractions of the piezoelectric and magnetostrictive phases are:

(39) $$\begin{array}{l} {}^{p}\nu =\frac{{}^{p}t}{t},\\ {}^{m}\nu =\frac{{}^{m}t}{t}. \end{array}$$

**Figure 5:**
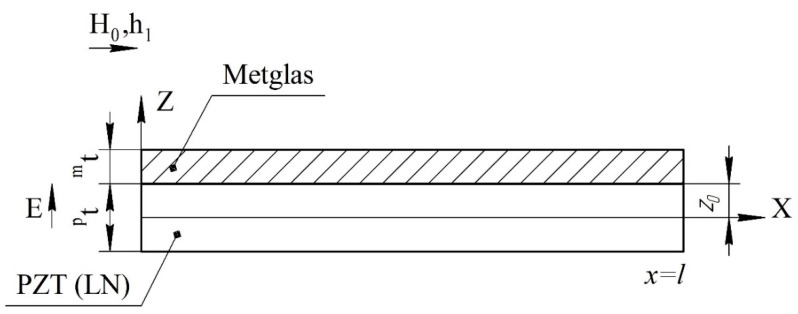
The position of the boundary between the piezoelectric and magnetostrictive phases relative to the neutral line in a two-layer composite.

The *X* axis will be drawn along the neutral line of the ME composite (Figure 5).

In the case of rigid connection between the components of the composite, we have:

(40) Sm1=Sp1=S1=−z∂2w∂x2,

where *w* is the transverse displacement.

The longitudinal component of the stress tensor and the third component of the electric stress vector of a piezoelectric phase are given by:

(41) T1p=c11DS1−h31D3

(42) $$E_{3}=-h_{31}S_{1}+\beta_{33}^{S}D_{3},$$

where $c_{11}^{D}$ is longitudinal component of the stiffness tensor at a constant electrical displacement; *h_31_* is piezoelectric coefficient at a constant longitudinal component of the strain tensor; $\beta_{33}^{S}$ is inverse permittivity at a constant longitudinal component of the strain tensor:

(43) c11D=(sp11E−d312ε33Tε0)−1h31=c11Dd31ε33Tε0β33S=1+h31d31ε33Tε0

where sp11E is compliance tensor component at constant electric field strength of the piezoelectric phases; $\varepsilon_{33}^{T}$ is transversal component of the relative permittivity tensor at a constant longitudinal component of the stress tensor.

Substituting Equation (40) in Equation (9), we get:

(44) Tm1=−zYmB∂2w∂x2−q¯11h1

The torque is:

(45) M=∫z0−tpz0bzTp1dz+∫z0z0+tmbzTm1dz=−b∂2w∂x2D−btp2〈h31〉D3−btm2〈q11〉h1,

where *b* is the sample width, *z*_0_ is position of the boundary between the piezoelectric and magnetostrictive phases relative to the neutral line, and:

(46) 〈h31〉=1tp2∫z0−tpz0zh31dz=2z0−tp2tph31〈q11〉=1tm2∫z0z0+tmzq¯11dz=q¯11(2z0+tm)2tm

*D= ^p^D + ^m^D* is full cylindrical stiffness of the composite:

(47) Dp=13c11Dtp(tp2−3tpz0+3z02)Dm=13YmBtm(tm2+3tmz0+3z02)

Then, the voltage across the piezoelectric phase is:

(48) U=∫z0−tpz0E3dz=tp2〈h31〉∂2w∂x2+tpβ33SD3

From Equation (48) we obtain the electric displacement in the piezoelectric phase:

(49) D3=Utpβ33S−tp〈h31〉β33S∂2w∂x2

Substituting the resulting expression in Equation (45):

(50) M=−bt3〈c11〉∂2w∂x2−btp〈h31〉β33SU−btm2〈q11〉h1,

where:

(51) 〈c11〉=1t3(D−tp3〈h31〉2β33S).

The position of the boundary between the piezoelectric and magnetostrictive phases relative to the neutral line *z*_0_ is determined from the minimum condition $\langle c_{11}\rangle$:

(52) z0=(c11Dpt2−YmBmt2)β33S−h312pt22(YmBmt+c11Dpt)β33S−h312pt

The shear force is:

(53) $$V=\frac{\partial M}{\partial x}=-bt^{3}\langle c_{11}\rangle \frac{\partial^{3}w}{\partial x^{3}}$$

The equation of bending vibrations can be written as:

(54) $$\rho bt\frac{\partial w^{2}}{\partial \tau^{2}}=\frac{\partial V}{\partial x}$$

Substituting Equation (53) in Equation (54), we obtain:

(55) $$t^{2}\langle c_{11}\rangle \frac{\partial^{4}w}{\partial x^{4}}+\rho \frac{\partial w^{2}}{\partial \tau^{2}}=0$$

Given that the time dependence of the shift is harmonic $w∼e^{i\omega t}$, the equation of bending vibrations can be written as:

(56) $$\begin{array}{l} \frac{\partial^{4}w}{\partial x^{4}}-k^{4}w=0,\\ k={\left(\frac{\rho }{t^{2}\langle c_{11}\rangle }\omega^{2}\right)}^{\frac{1}{4}}. \end{array}$$

The general solution of the motion equation is:

(57) $$w=C_{1}\cosh\left(kx\right)+C_{2}\sinh\left(kx\right)+C_{3}\cos\left(kx\right)+C_{4}\sin\left(kx\right),$$

where *C*_1_, C_2_, C_3_, C_4_ are unknown constants.

The open circuit condition is:

(58) $$\int_{0}^{l}D_{3}dx=0$$

Integrating Equation (48) over *x*, we obtain:

(59) Ul=tp2〈h31〉∂w∂x|0l=tp2〈h31〉k[C1r2+C2(r1−1)−C3r4+C4(r3−1)],

where:

(60) $$\begin{array}{l} r_{1}=\cosh\left(kl\right)\\ r_{2}=\sinh\left(kl\right)\\ r_{3}=\cos\left(kl\right)\\ r_{4}=\sin\left(kl\right) \end{array}$$

Free Clamping of Both Ends of the ME Composite.

The boundary conditions for free ends of the beam are given by:

(61) $$\begin{array}{l} V\left(0\right)=0,\\ M\left(0\right)=0,\\ V\left(l\right)=0,\\ M\left(l\right)=0. \end{array}$$

Combining Equation (61) with Equation (59), we obtain a linear system of five inhomogeneous algebraic equations for five unknowns *C*_1_, C_2_, C_3_, C_4_, *U*:

(62) C2−C4=0−t3〈c11〉k2(C1−C3)−tp〈h31〉β33SU−tm2〈q11〉h1=0C1r2+C2r1+C3r4−C4r3=0−t3〈c11〉k2(C1r1+C2r2−C3r3−C4r4)−tp〈h31〉β33SU−tm2〈q11〉h1=0Ul=tp2〈h31〉k[C1r2+C2(r1−1)−C3r4+C4(r3−1)]

We solve this system by considering the fact that:

(63) $$\begin{array}{l} r_{1}^{2}-r_{2}^{2}=1\\ r_{3}^{2}+r_{4}^{2}=1 \end{array}$$

The voltage across the piezoelectric is given by the following equation:

(64) U=2tm2tp2〈q11〉〈h31〉β33S(r1r4+r2r3−r2−r4)〈c11〉klt3β33S(1−r1r3)−2tp3〈h31〉2(r1r4+r2r3−r2−r4)h1.

As a result, the ME voltage coefficient is obtained in the form:

(65) αE=E¯3h1=2tm2tp2〈q11〉〈h31〉β33S(r1r4+r2r3−r2−r4)t[〈c11〉klt3β33S(1−r1r3)−2tp3〈h31〉2(r1r4+r2r3−r2−r4)].

**Figure 6:**
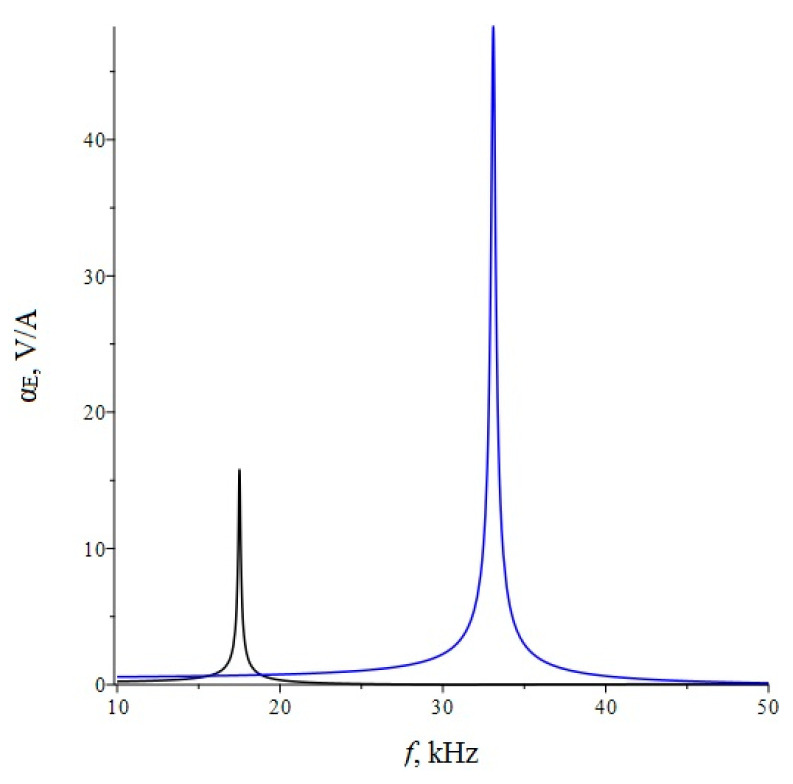
Theoretical dependence of the ME voltage coefficient on the frequency of the alternating magnetic field. Black color of the line is PZT, blue is LN cut y + 128°.

Below, Figure 6 shows the dependence of the ME voltage coefficient on the frequency of the alternating magnetic field for two cases, when PZT and a cut of lithium niobate y + 128° are taken as the piezoelectric phase. Metglas is taken as the magnetostrictive phase. For the calculation, the following thicknesses of Metglas *^m^t* = 29 µm and piezoelectric *^p^t* = 0.5 mm are taken, and the length of ME composite is l = 10 mm. To take into account losses in the calculation, it is assumed: *ω* = 2*π*(1 + (1/2*Q*)*i*)*f*, where *Q* is the quality factor of the resonant system. The value of the quality factor was taken to be the same as for the longitudinal mode, *Q* = 130.

The fundamental resonant frequency for this case is:

(66) $$\begin{array}{l} f_{r}=\frac{\chi^{2}t}{2\pi l^{2}}\sqrt{\frac{\langle c_{11}\rangle }{\rho }}\\ \chi =473 \end{array}$$

Cantilever Clamping of ME Composite.

The boundary conditions for this case:

(67) $$\begin{array}{l} w\left(0\right)=0,\\ \frac{\partial w}{\partial x}\left(0\right)=0,\\ V\left(l\right)=0,\\ M\left(l\right)=0. \end{array}$$

The general solution of the equation of motion is:

(68) $$w=C_{1}\cosh\left(kx\right)+C_{2}\sinh\left(kx\right)+C_{3}\cos\left(kx\right)+C_{4}\sin\left(kx\right)$$

The linear system of five inhomogeneous algebraic equations for five unknowns *C*_1_, C_2_, C_3_, C_4_, *U*:

(69) C1+C3=0C2+C4=0C1r2+C2r1+C3r4−C4r3=0−t3〈c11〉k2(C1−C3)−tp〈h31〉β33SU−tm2〈q11〉h1=0−t3〈c11〉k2(C1r1+C2r2−C3r3−C4r4)−tp〈h31〉β33SU−tm2〈q11〉h1=0Ul=tp2〈h31〉k[C1r2+C2(r1−1)−C3r4+C4(r3−1)]

We solve this system, considering the fact that:

(70) $$\begin{array}{l} r_{1}^{2}-r_{2}^{2}=1\\ r_{3}^{2}+r_{4}^{2}=1 \end{array}$$

The voltage across the piezoelectric is given by:

(71) U=−tm2tp2〈q11〉〈h31〉β33S(r1r4+r2r3)〈c11〉klt3β33S(1+r1r3)+tp3〈h31〉2(r1r4+r2r3)h1.

As a result, the ME voltage coefficient is obtained as:

(72) αE=E3¯h1=−tm2tp2〈q11〉〈h31〉β33S(r1r4+r2r3)[〈c11〉klt3β33S(1+r1r3)+tp3〈h31〉2(r1r4+r2r3)]t.

**Figure 7:**
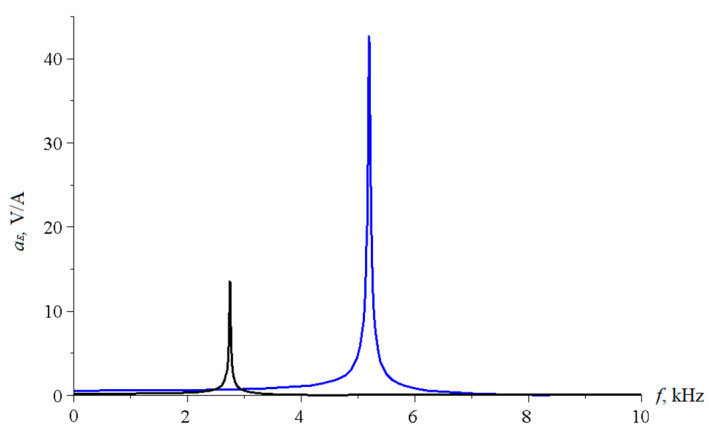
Theoretical dependence of the ME voltage coefficient on the frequency of the alternating magnetic field. Black color of the line is PZT, blue is LN cut y + 128°.

Below, Figure 7 shows the dependence of the ME voltage coefficient on the frequency of the alternating magnetic field for two cases, when PZT and a cut of lithium niobate y + 128° are taken as the piezoelectric phase. Metglas is taken as the magnetostrictive phase. For the calculation, the following thicknesses of Metglas *^m^t* = 29 µm and piezoelectric *^p^t* = 0.5 mm are taken, length of ME composite is l = 10 mm. To take into account losses in the calculation, it is assumed: *ω* = 2*π*(1 + (1/2*Q*)*i*)*f*, where *Q* is the quality factor of the resonant system. The value of the quality factor was taken the same as for the longitudinal mode *Q* = 130.

The fundamental resonant frequency for this case is:

(73) $$\begin{array}{l} f_{r}=\frac{\chi^{2}t}{2\pi l^{2}}\sqrt{\frac{\langle c_{11}\rangle }{\rho }}\\ \chi =1875 \end{array}$$

In [11,12], the theory of the bending mode of the ME effect in the EMR region was considered, based on the hypothesis that the electric field strength in the piezoelectric phase is independent of the coordinate along the thickness of an asymmetric magnetostrictive-piezoelectric composite, and its satisfactory agreement with experimental data was shown. The theory of the same phenomenon based on a more plausible hypothesis of independence of the electric displacement in the piezoelectric phase from the coordinate along the thickness of an asymmetric magnetostrictive piezoelectric composite was considered in [13], and it showed good agreement with the experiment. However, in this work, the corresponding theory is presented very briefly. In our article, we describe this theory in as much detail as possible for a better understanding and ease of application, if necessary.

#### 2.2.3. Quasi-Static Mode

In [14,15], the theory of the longitudinal and bending modes of the ME effect in the quasi-static mode for an asymmetric magnetostrictive-piezoelectric structure was considered. Separate expressions are found for the contributions of the planar and bending modes, and then the full expression. In our article, we start immediately from general expressions that consider the planar and flexural modes and obtain the result. We do this in as much detail as possible to facilitate understanding and ease of application of this theory if necessary.

In the quasi-static mode, Equation (18) is given by:

(74) $$\frac{\partial T_{1}}{\partial x}=0$$

This means that *T*_1_ must not depend on *x*. It is obvious that *S*_1_ must not depend on *x* either. Since both the longitudinal and bending modes are excited in the asymmetric ME structure in the quasi-static mode:

(75) $$S_{1}=A+zB,$$

where *A,* *B* are unknown constants.

Substituting Equation (75) in Equation (9) and Equation (41), also considering that due to the open circuit condition *D*_3_ = 0, we obtain:

(76) Tm1=YmB(A+zB)−q¯11h1=YmBA−q¯11h1+YmBzB

(77) Tp1=c11D(A+zB)=c11DA+c11DzB

The first condition for the static equilibrium of the ME composite is the equality to zero of the total longitudinal force is given by:

(78) ∫z0−tpz0Tp1dz+∫z0z0+tmTm1dz=0

Substituting Equations (76) and (77) in Equation (78), we obtain:

(79) tpc11DA+12c11DBtp(2z0−tp)+(YmBA−q¯11h1)tm+12YmBBtm(2z0+tm)=0.

The second condition for the static equilibrium of the ME composite is the zero total moment is given by:

(80) ∫z0−tpz0zTp1dz+∫z0z0+tmzTm1dz=0

Substituting Equations (76) and (77) in Equation (80), we obtain:

(81) 12c11DAtp(2z0−tp)+13c11DBtp(tp2−3tpz0+3z02)++12(YmBA−q¯11h1)tm(2z0+tm)+13YmBBtm(tm2+3tmz0+3z02)=0

The Equations (79) and (81) form a linear inhomogeneous system of two equations with two unknowns *A*, *B*. Solving them, we obtain *A* and *B* as:

(82) A=(3c11Dtmtp2−6c11Dtmtpz0−6c11Dtp2z0+YmBtm3)tmq¯11h1(c11D)2tp4+4c11DYmBtptm3+6c11DYmBtp2tm2+4c11DYmBtmtp3+(YmB)2tm4B=6c11Dtmtp(tm+tp)q¯11h1(c11D)2tp4+4c11DYmBtptm3+6c11DYmBtp2tm2+4c11DYmBtmtp3+(YmB)2tm4

Substituting Equation (75) in Equation (42), and considering that due to the open circuit condition *D*_3_ = 0, we get *E*_3_:

(83) $$E_{3}=-h_{31}\left(A+zB\right).$$

Then, the voltage across the piezoelectric is:

(84) U=∫z0−tpz0−h31(A+zB)dz=−h31[Atp+12Btp(2z0−tp)].

Substituting Equation (82) in Equation (84), we obtain:

(85) U=−tmtp(c11Dtp3+YmBtm3)h31q¯11h1(c11D)2tp4+4c11DYmBtptm3+6c11DYmBtp2tm2+4c11DYmBtmtp3+(YmB)2tm4

From Equation (85) we find the ME voltage coefficient as:

(86) αE=−tmtp(c11Dtp3+YmBtm3)h31q¯11[(c11D)2tp4+4c11DYmBtptm3+6c11DYmBtp2tm2+4c11DYmBtmtp3+(YmB)2tm4](tm+tp)

**Figure 8:**
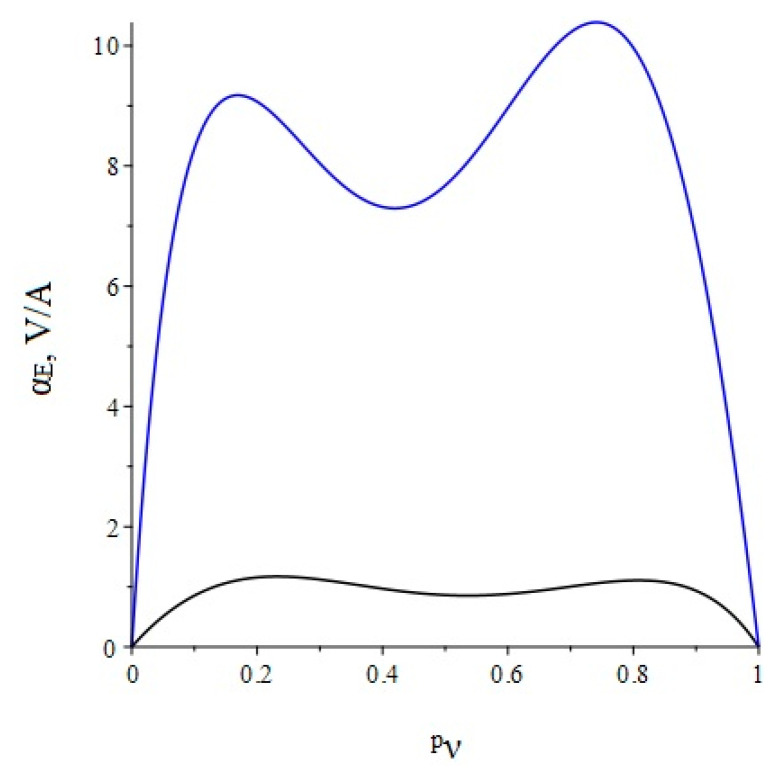
Theoretical dependence of the ME voltage coefficient on the volume fraction of the piezoelectric. Black color of the line is PZT, blue is LN cut y + 128°.

Below, Figure 8 shows the dependence of the ME voltage coefficient on the volume fraction of the piezoelectric for two cases, when PZT and a cut of lithium niobate y + 128° are taken as the piezoelectric phase. Metglas is taken as the magnetostrictive phase.

## 3. Longitudinal-Shear and Torsional Modes

### 3.1. Symmetrical ME Structure

In a symmetric ME structure, excitation of the torsional mode of the ME effect is impossible. Therefore, we first consider the general case of a longitudinal-shear mode for an arbitrary frequency of an alternating magnetic field, which also includes the resonant mode, and the expression for the ME voltage coefficient for the quasi-static case will be obtained from the general expression, assuming the frequency f is equal to zero.

#### 3.1.1. Resonance Mode

**Figure 9:**
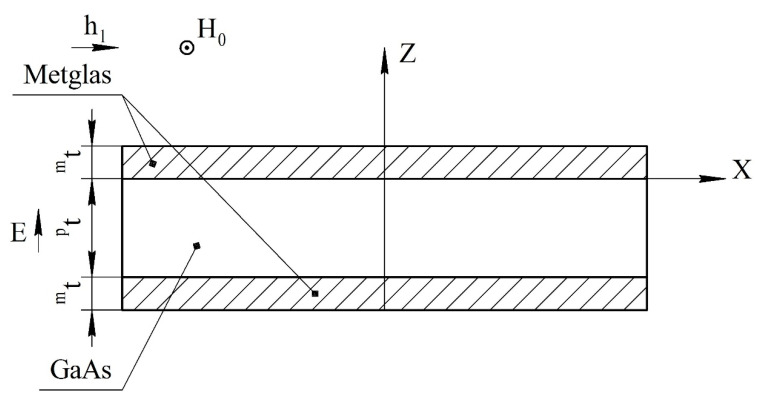
Symmetric tri-layer magnetoelectric composite for calculations of longitudinal-shear mode. In contrast to Figure 1, the magnetic fields are mutually perpendicular to each other.

We consider a magnetoelectric composite as a thin narrow plate. Layers of the magnetostrictive phase of the same thickness are above and below the piezoelectric layer. The ME structure created in this way is symmetrical. The *X* axis is directed along the length of the plate, and the *Z* axis is perpendicular to the sample plane, as in Figure 9.

We consider small longitudinal-shear mechanical oscillations in a composite under the influence of a small external variable magnetic field. The AC magnetic field is directed along the *X* axis, and the DC magnetic field is directed along the *Y* axis, then:

(87) $$h_{1}\left(t\right)=h_{1}e^{i\omega t}.$$

The material equation for the piezoelectric layer is given by:

(88) Sp6=d36E3+sp66Tp6,

where *^p^S*_6_ is shear strain tensor component of piezoelectric phase; *d*_36_ is piezoelectric coefficient; *^p^s*_66_ is shear compliance tensor component of the piezoelectric phase; *^p^T*_6_ is the shear stress tensor component of the piezoelectric phase.

The shear component of the stress tensor in a piezoelectric can be expressed as:

(89) Tp6=1sp66Sp6−d36sp66E3

The shear component of the stress tensor in the magnetostrictive phase has the form:

(90) mT6=Gm(Sm6−q16h1)=GmSm6−q¯16h1,

where:

(91) q¯16=Gmq16,

where *^m^S*_6_ is shear strain tensor component of magnetostrictive phase, *^m^G* is shift modulus in the magnetostrictive phase, and *q*_16_ is corresponding pseudo-piezomagnetic coefficient.

In accordance with the condition of the problem for longitudinal-shear mode is:

(92) Sm6=Sp6=S6=∂Ux∂y+∂Uy∂x=∂Uy∂x

Shear component of the composite stress tensor is:

(93) T6=νmTm6+νpTp6=c66S6−νmq¯16h1−νpd36sp66E3,

where volume fractions of the piezoelectric and magnetostrictive phases are:

(94) νp=tptp+2tmνm=2tmtp+2tm

The effective shear composite stiffness coefficient is:

(95) c66=νpsp66+νmGm.

Composite effective density is given by:

(96) ρ=νpρp+νmρm.

Consider the motion equation for deformations is:

(97) $$\rho \frac{\partial^{2}U_{y}}{\partial t^{2}}=\frac{\partial T_{6}}{\partial x}.$$

Substituting Equation (90) in Equation (94), we get:

(98) $$-\rho \omega^{2}U_{y}=c_{66}\frac{\partial^{2}U_{y}}{\partial x^{2}}$$

The solution of this equation is obtained as:

(99) $$U_{y}=A\cos\left(kx\right)+B\sin\left(kx\right),$$

where the wave number is:

(100) $$k=\sqrt{\frac{\rho }{c_{66}}}\omega ,$$

and *A*, *B* are unknown constants.

Then:

(101) $$S_{6}=\frac{\partial U_{y}}{\partial x}=\left(B\cos\left(kx\right)-A\sin\left(kx\right)\right)k,$$

(102) T6=c66(Bcos(kx)−Asin(kx))k−νmq¯16h1−νpd36sp66E3.

To obtain the constants *A* and *B*, we use the equilibrium conditions for a free sample:

(103) $$\begin{array}{l} {T_{6}|}_{x=-\frac{l}{2}}=0\\ {T_{6}|}_{x=\frac{l}{2}}=0 \end{array}$$

Substituting Equation (103) in Equation (102), we get:

(104) c66(Bcos(η)+Asin(η))k−νmq¯16h1−νpd36sp66E3=0c66(Bcos(η)−Asin(η))k−νmq¯16h1−νpd36sp66E3=0

where:

(105) $$\eta =\frac{kl}{2},$$

As a result, we get:

(106) A=0B=νmq¯16sp66h1+νpd36E3sp66c66kcos(η)

The transverse component of the electric displacement vector can be obtained from:

(107) D3=εε0E3+d36Tp6=[εε0−d362sp66]E3+d36sp66S6.

We can find the transverse component of the electric field strength vector from the condition that the electric induction flux through the interface between the upper layer of the magnetostrictive phase and the piezoelectric are equal to zero:

(108) $$\int_{-\frac{l}{2}}^{\frac{l}{2}}D_{3}dx=0.$$

Substituting Equation (104) in Equation (105), we get:

(109) [εε0−d362sp66]E3l+2d36sp66Bsin(η)=0

Substituting Equation (103) in Equation (106):

(110) [εε0−d362sp66]E3l+2d36sp66sin(η)νmq¯16sp66h1+νpd36E3sp66c66kcos(η)=0.

From Equation (110), *E*_3_ is obtained as:

(111) E3=−νmq¯16d36sp66tan(η)εε0sp662c66η+d362[νptan(η)−c66sp66η]h1.

As the electric field exists only in the piezoelectric phase, the voltage is given by the following equation:

(112) U=E3tp.

Average electric field strength in ME composite is:

(113) E¯=Utm+tp=νpE3

Then, the ME voltage coefficient is obtained as:

(114) αE=E¯h1=−νmνpq¯16d36sp66tan(η)εε0sp662c66η+d362[νptan(η)−c66sp66η].

**Figure 10:**
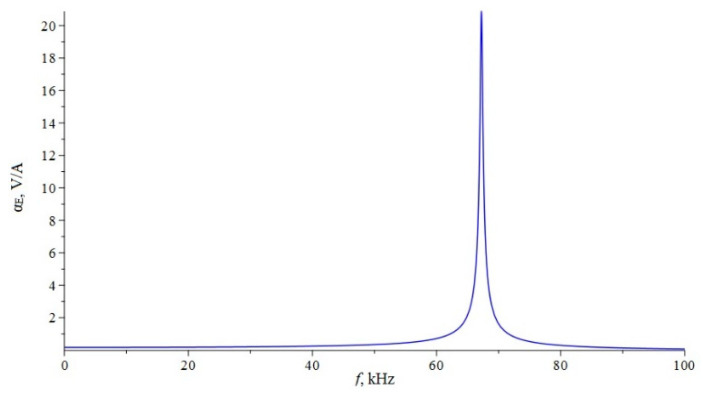
Theoretical dependence of the ME voltage coefficient on the frequency of the alternating magnetic field for symmetrical ME structure Metglas/GaAs of the longitudinal-shear mode.

Below, Figure 10 shows the dependence of the ME voltage coefficient on the frequency of the alternating magnetic field. To take into account losses in the calculation, it is assumed: *ω* = 2*π*(1 + (1/2*Q*)*i*)*f*, where *Q* is the quality factor of the resonant system. In the calculation, the following material parameters of the initial components were used: for Metglas: *^m^ρ* = 7180 kg/m^3^, *^m^G* = 3.85 × 10^10^ Pa, *q*_16_ = 1.0 × 10^−9^ m/A, *^m^t* = 29 µm; for gallium arsenide (GaAs) [18] *^p^ρ* = 5320 kg/m^3^, shift modulus *^p^G* = 5.94 × 10^10^ Pa, *ε* = 12.9, *d*_36_ = −2.69 × 10^−12^ m/V, *^p^t* = 2 × 10^−4^ m. Sample length l = 2.3 × 10^−2^ m, width *b* = 3 × 10^−4^ m. The value of the quality factor was *Q* = 300.

The fundamental resonant frequency for this case is:

(115) $$f_{r}=\frac{1}{2l}\sqrt{\frac{c_{66}}{\rho }}$$

#### 3.1.2. Quasi-Static Mode

Assuming in Equation (114) the frequency f equal to zero, we obtain:

(116) αE=E¯h1=−νmνpq¯16d36sp66εε0sp662c66+d362[νp−c66sp66].

**Figure 11:**
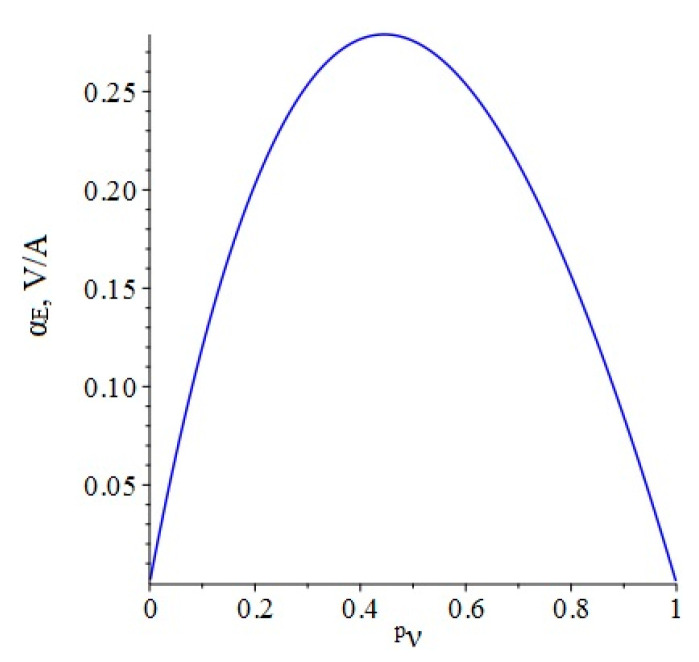
Theoretical dependence of the ME voltage coefficient on the volume fraction of the piezoelectric material for ME structure Metglas/GaAs of the longitudinal-shear mode.

Below, Figure 11 shows the dependence of the ME voltage coefficient on the volume fraction of the piezoelectric, when GaAs are taken as the piezoelectric phase. Metglas is taken as the magnetostrictive phase.

### 3.2. Asymmetric ME Structure

#### 3.2.1. Resonance Regime for the Longitudinal-Shear Mode

**Figure 12:**
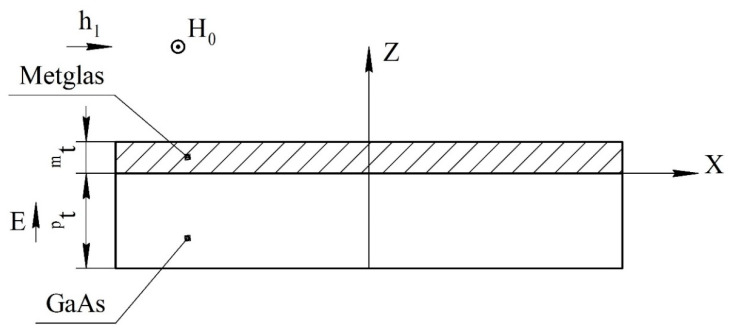
Asymmetric two-layer magnetoelectric composite for calculation of longitudinal-shear mode. All designations are the same as in Figure 9.

For an asymmetric ME structure in the resonant mode of the longitudinal-shear ME mode, the voltage coefficient can be found from Equation (114). Only in Equation (94) is it necessary to remove the number 2 before *^m^t*. The fundamental resonant frequency for this case can be found in Equation (115). The ME structure shown at Figure 12. 

**Figure 13:**
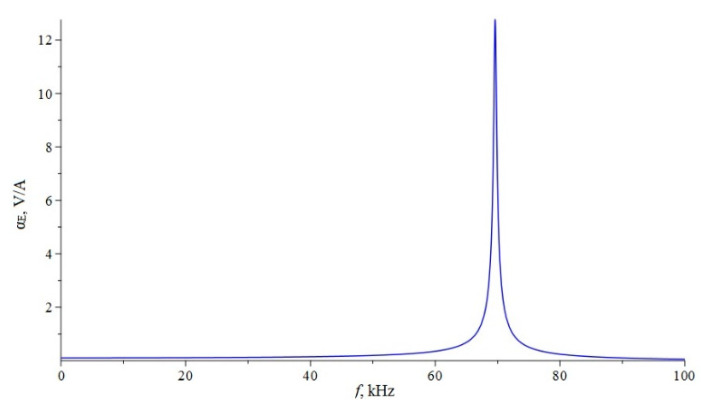
Theoretical dependence of the ME voltage coefficient on the frequency of the alternating magnetic field for asymmetrical ME structure Metglas/GaAs of the longitudinal-shear mode.

Below, Figure 13 shows the dependence of the ME voltage coefficient on the frequency of the alternating magnetic field. To take into account losses in the calculation, it is assumed: *ω* = 2*π*(1 + (1/2*Q*)*i*)*f*, where *Q* is the quality factor of the resonant system.

#### 3.2.2. Resonant Regime for the Torsional Mode

In [16,17], the theory of the torsional mode of the ME effect in the EMR region was considered for an asymmetric magnetostrictive-piezoelectric structure. However, since the torsion of the structure was considered around the axis passing along the width of the structure, the numerical values of the ME stress coefficient turned out to be too small for the torsional mode to be seen against the background of a relatively large longitudinal shear mode. In this article, we consider the torsion of an ME structure around an axis running along the length of the structure. This made it possible to obtain relatively large values of the ME voltage coefficient.

**Figure 14:**
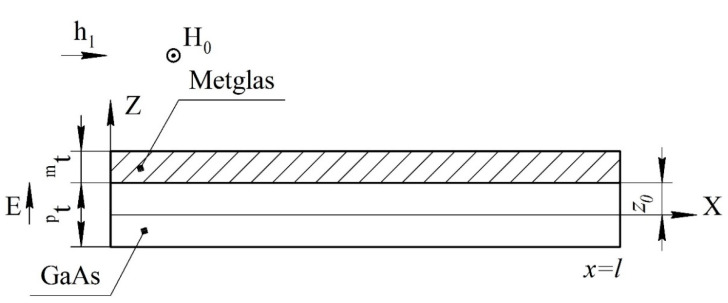
Asymmetric two-layer magnetoelectric composite for calculation of torsional mode.

Draw the *X* axis along the length of the sample in the corresponding plane of symmetry of the sample, and the *Y* axis along the axis of rotation of the composite beam during torsional vibrations in the direction of the sample width as in Figure 14.

The AC magnetic field is directed along the *X* axis, and the DC magnetic field is directed along the *Y* axis.

The full thickness of the composite:

(117) t=tp+tm.

Shear components of the strain tensor are:

(118) $$\begin{array}{l} S_{5}=y\frac{\partial \theta }{\partial x}\\ S_{6}=-z\frac{\partial \theta }{\partial x} \end{array}$$

where *θ*—the twist angle.

Material equations for a piezoelectric phase:

(119) S5=1GpTp5S6=1GpTp6+d36Ep3

where *^p^E_3_* is electric field intensity in a piezoelectric.

From Equation (115), we find the tangent components of the stress tensor for the piezoelectric:

(120) pT5=GpS5=Gpy∂θ∂x

(121) pT6=Gp(S6−d36Ep3)=−Gpz∂θ∂x−d36GpEp3.

Material equations for a ferromagnet:

(122) S5=1GmTm5S6=1GmTm6+q16h1

From Equation (118), the tangent components of the stress tensor of the magnetostrictive phase:

(123) mT5=GmS5=Gmy∂θ∂xmT6=Gm(S6−q16h2)=−Gmz∂θ∂x−q¯16h1

where:

(124) q¯16=Gmq16.

The electrical displacement in piezoelectric is equal:

(125) D3=d36Tp6+εε0Ep3=−d36pGz∂θ∂x+(εε0−Gpd362)Ep3.

From Equation (121), the electric field *^p^E_3_* is obtained as:

(126) Ep3=h36z∂θ∂x+β33SD3,

where:

(127) h36=d36Gpεε0−Gpd362β33S=1εε0−Gpd362

Substituting in Equation (117), we get:

(128) pT6=−GpDz∂θ∂x−h36D3,

where shear modulus at constant electrical displacement of the piezoelectric phase:

(129) GpD=εε0Gpεε0−Gpd362.

The torque of composite is:

(130) M=∫−b2b2dy∫z0−tpz0(yTp5−zTp6)dz+∫−b2b2dy∫z0z0+tm(yTm5−zTm6)dz==∫−b2b2dy∫z0−tpz0(yGpy∂θ∂x−z(−GpDz∂θ∂x−h36D3))dz++∫−b2b2dy∫z0z0+tm(yGmy∂θ∂x−z(−Gmz∂θ∂x−q¯16h1))dz=K0∂θ∂x+btp2〈h36〉D3+btm2〈q16〉h1

where:

(131) K=Kp+KmKp=13GpD(z03−(z0−tp)3)b+112Gptpb3Km=GmIm

where *z*_0_ is the position of the interface between the piezoelectric and magnetostrictive phases relative to the axis of rotation of the composite beam, and polar moment of a ferromagnet:

(132) Im=13((z0+tm)3−z03)b+112tmb3

(133) 〈h36〉=1tp2∫z0−tpz0zh36dz=h36(2z0−tp)2tp〈q16〉=1tm2∫z0z0+tmzq¯16dz=q¯16(2z0+tm)2tm

The voltage across the piezoelectric phase is given by the following equation:

(134) U=∫z0−tpz0Ep3dz=∫z0−tpz0(h36z∂θ∂x+β33SD3)dz=tp2〈h36〉∂θ∂x+tpβ33SD3.

From Equation (134), the electric displacement in the piezoelectric phase is:

(135) D3=Utpβ33S−tp〈h36〉β33S∂θ∂x

Substituting in Equation (126), we find:

(136) M=−bt3〈G〉∂θ∂y−btp〈h36〉〈β33S〉U+btm2〈q16〉h1,

where effective shear modulus of the sample:

(137) 〈G〉=1bt3(K−btp3〈h36〉2〈β33S〉).

The position of the interface between the piezoelectric and magnetostrictive phases relative to the axis of rotation of the composite beam *z*_0_ is determined from the condition of the minimum effective shear modulus of the sample 〈G〉:

(138) z0=GpDpt2β33S−Gmmt2β33S−h362pt22(Gmmtβ33S+GpDptβ33S−h362pt).

The torsional vibrations are:

(139) $$J\frac{\partial \theta^{2}}{\partial \tau^{2}}=\frac{\partial M}{\partial x},$$

where the moment of inertia of the sample per unit width:

(140) J=ρpIp+ρmIm,

where the polar moment of the piezoelectric is:

(141) Ip=13(z03−(z0−tp)3)b+112tpb3.

Substituting Equation (133) in Equation (136), we get:

(142) $$J\frac{\partial \theta^{2}}{\partial \tau^{2}}=-bt^{3}\langle G\rangle \frac{\partial^{2}\theta }{\partial x^{2}}.$$

The dependence of the twist angle on time is harmonic $\theta ∼e^{i\omega t}$, therefore:

(143) $$\frac{\partial^{2}\theta }{\partial x^{2}}+k^{2}\theta =0,$$

where the wave number is:

(144) $$k=\omega \sqrt{\frac{J}{bt^{3}\langle G\rangle }}.$$

The general solution of the Equation (140) is:

(145) θ=Acos(kx)+Bsin(kx),

where *A*, *B* are unknown constants.

The open circuit condition is:

(146) $$\int_{-\frac{l}{2}}^{\frac{l}{2}}D_{3}dx=0.$$

Then, we integrate Equation (135) over *x*:

(147) Ul=tp2〈h36〉θ|−l2l2=2tp2〈h36〉Bsinη,

where:

(148) $$\eta =\frac{kl}{2}.$$

Boundary conditions for a free sample are:

(149) $$\begin{array}{l} M\left(\frac{l}{2}\right)=0\\ M\left(-\frac{l}{2}\right)=0 \end{array}$$

Combining boundary conditions Equation (146) with Equation (144), we obtain a linear system of three inhomogeneous algebraic equations with three unknowns, *A*, *B*, *U*:

(150) −kbt3〈G〉(Bcosη−Asinη)−btp〈h36〉β33SU+btm2〈q16〉h1=0−kbt3〈G〉(Bcosη+Asinη)−btp〈h36〉β33SU+btm2〈q16〉h1=0Ul=2tp2〈h36〉Bsinη

As a result, the ME voltage coefficient is obtained as:

(151) αE=2tp2tm2〈h36〉〈q16〉β33Stanηt(klt3〈G〉β33S+2〈h36〉2tp3tanη).

**Figure 15:**
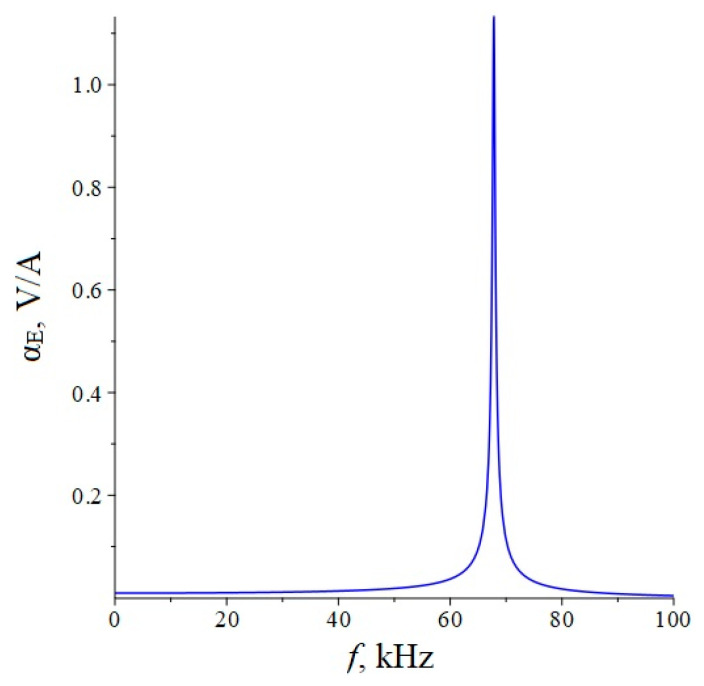
Theoretical dependence of the ME voltage coefficient on the frequency of the alternating magnetic field for ME structure Metglas/GaAs of the torsional mode.

Figure 15 shows the dependence of the ME voltage coefficient on the frequency of the alternating magnetic field. To take into account losses in the calculation, it is assumed: *ω* = 2*π*(1 + (1/2*Q*)*i*)*f*, where *Q* is the quality factor of the resonant system. In the calculation, the same material parameters were used as for the longitudinal-shear mode.

The fundamental resonant frequency for this case is:

(152) $$f_{r}=\frac{1}{2l}\sqrt{\frac{bt^{3}\langle G\rangle }{J}}.$$

#### 3.2.3. Quasi-Static Mode

In the quasi-static mode, there are no vibrations along the length of the composite. This means that *S*_5_ and *S*_6_ must not depend on x. Since both the longitudinal-shear and torsional modes are excited in the asymmetric ME structure in the quasistatic mode, then:

(153) $$\begin{array}{l} S_{5}=yB\\ S_{6}=A-zB \end{array}$$

where *A*, *B* are unknown constants.

Substituting Equation (153) in Equations (120), (123), and (128), also considering that due to the open circuit condition *D*_3_ = 0, we obtain:

(154) mT5=GmS5=GmyBmT6=Gm(S6−q16h1)=GmA−GmzB−q¯16h1

(155) pT5=GpS5=GpyBpT6=GpDA−GpDzB

The first condition for the static equilibrium of the ME composite is the total tangential force on the site perpendicular to the *x* axis along the y axis is equal to zero and is given by the following equation:

(156) ∫z0−tpz0Tp6dz+∫z0z0+tmTm6dz=0.

Substituting Equations (154) and (155) in Equation (156), we get:

(157) −12GpDB[z02−(z0−tp)2]+GpDAtp−12GmB[(z0+tm)2−z02]+Gmmt(A−q16h1)=0

The second condition for the static equilibrium of the ME composite is the zero torque and given by:

(158) ∫−b2b2dy∫z0−tpz0(yTp5−zTp6)dz+∫−b2b2dx∫z0z0+tm(yTm5−zTm6)dz=0

Substituting Equations (154) and (155) in Equation (158), we get:

(159) 13GpDB[z03−(z0−tp)3]−112GpDAb[z02−(z0−tp)2]+112BGptpb3+13GmBb[(z0+mt)3−z03]−−12Gmb(A−q16h1)[(z0+tm)2−z02]+112GmBmtb3=0

The Equations (153) and (155) form a linear inhomogeneous system of two equations with two unknowns *A*, *B*. Solving it, we find *A* and *B*:

(160) A=Gmmtq16h1[b2(Gmmt+Gppt)+Gmmt3+Gmmtb2(mtGm+Gppt+GpDpt)+Gm2mt4++GpD(3tp2mt−6z0ptmt+4tp3−6z0pt2)]+GpDpt(Gpptb2+4Gmmt3+6Gmmt2pt+4Gmmtpt2+GpDpt3)B=−6Gmmtq16h1pt⋅Gmmtb2(mtGm+Gppt+GpDpt)+Gm2mt4+⋅(mt+tp)+GpDpt(Gpptb2+4Gmmt3+6Gmmt2pt+4Gmmtpt2+GpDpt3)

Substituting Equation (153) in Equation (126), and considering that due to the open circuit condition *D*_3_ = 0, *E*_3_ is obtained as:

(161) $$E_{3}=-h_{36}(A-zB).$$

The voltage across the piezoelectric:

(162) U=∫z0−tpz0−h36(A−zB)dz=−h36[Atp+12Btp(2z0−tp)].

Substituting Equation (156) in Equation (158), we get:

(163) U=h1tptmGmq16h36[GpDtp3+Gmtm3+Gm2tm2(b2+tm2)+GpDtp2(GpDtp2+6tm2Gm+4tmGmtp+b2Gp)++b2(Gptp+Gmtm)]+Gmtptm(b2Gp+4GpDtm2+GpDb2)

From Equation (159) we find the ME voltage coefficient:

(164) αE=tptmGmq16h36[GpDtp3+Gmtm3+[Gm2tm2(b2+tm2)+GpDtp2(GpDtp2+6tm2Gm+4tmGmtp+b2Gp)++b2(Gptp+Gmtm)]+Gmtptm(b2Gp+4GpDtm2+GpDb2)](tp+tm)

**Figure 16:**
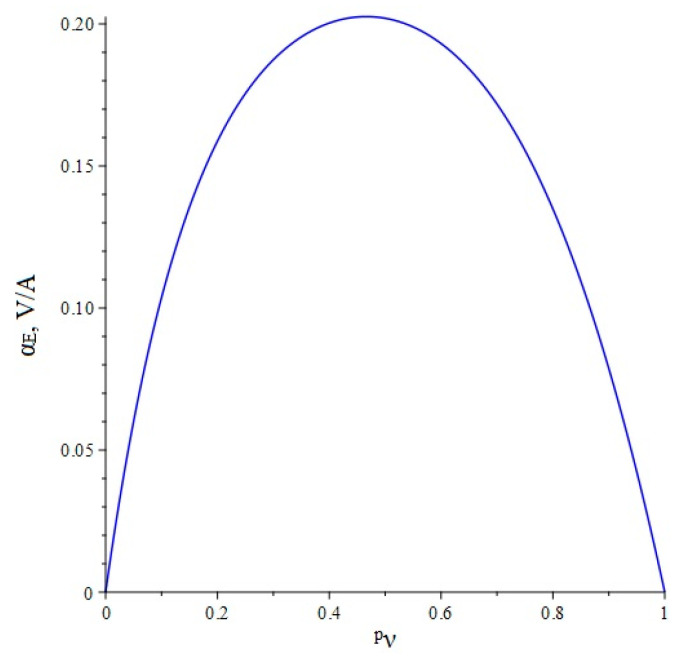
Theoretical dependence of the ME voltage coefficient on the volume fraction of the piezoelectric material for the asymmetric ME structure Metglas/GaAs for the quasi-static mode of the torsional and longitudinal-shear modes.

Below, Figure 16 shows the dependence of the ME voltage coefficient on the volume fraction of the piezoelectric, when GaAs are taken as the piezoelectric phase. Metglas is taken as the magnetostrictive phase. The material parameters of the ME structure are the same as for the calculation of the longitudinal shear mode.

As can be seen from the comparison of Figure 13 and Figure 15, the ME voltage coefficient in the EMR regime for the longitudinal-shear mode is several times larger than for the torsional mode. Therefore, it is quite natural that in the quasistatic mode the torsional mode does not make a very significant contribution, and the ME voltage coefficient in the quasistatic mode is mainly determined by the contribution of the longitudinal-shear mode.

### 3.3. ME Structure Based on Bimorph Lithium Niobate

#### 3.3.1. Resonant Regime for the Torsional Mode

**Figure 17:**
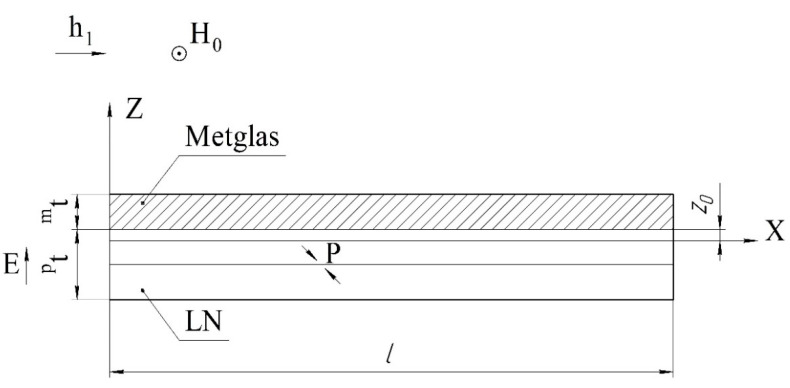
Asymmetric magnetoelectric composite with bimorth LiNbO_3_ layer at torsional vibrations.

Draw the *X* axis along the axis of rotation of the composite beam during torsional vibrations in the direction of the length of the sample, and the *Y* axis along the width of the sample in the corresponding plane of symmetry of the sample, as in Figure 17.

The AC magnetic field is directed along the *X* axis, and the DC magnetic field is directed along the *Y* axis.

The shear components of the strain tensor are given by:

(165) $$\begin{array}{l} S_{5}=y\frac{\partial \alpha }{\partial x}\\ S_{6}=-z\frac{\partial \alpha }{\partial x} \end{array}$$

where *α*—the twist angle.

Material equations for a ferromagnetic phase:

(166) S5=1GmTm5S6=1GmTm6+q16h1

From Equation (162), we find the tangent components of the stress tensor for the magnetostrictive phase:

(167) mT5=GmS5=Gmy∂α∂xmT6=Gm(S6−q16h1)=−Gmz∂α∂x−q¯16h1

where:

(168) q¯16=Gmq16.

Material equations for piezoelectric:

(169) Tp5=cp55ES5+cp56ES6−e35Ep3Tp6=cp56ES5+cp66ES6−e36Ep3D3=e35S5+e36S6+ε33ε0Ep3

where cp55E, cp56E, cp66E are shear components at a constant electric field strength of the stiffness tensor of the piezoelectric phase, e_35_, e_36_ are piezoelectric coefficients at constant electric field strength.

Express *^p^T_5_*, *^p^T_6_*, *^p^E_3_* from Equation (169):

(170) Tp5=cp55DS5+cp56DS6−h35D3Tp6=cp56DS5+cp66DS6−h36D3Ep3=−h35S5−h36S6+β33SD3=−h35y∂α∂x+h36z∂α∂x+β33SD3

where cp55D, cp56D, cp66D are shear components at a constant electrical displacement of the stiffness tensor of the piezoelectric phase, *h_35_*, *h_36_* are piezoelectric coefficients at constant shear components of the strain tensor:

(171) cp55D=cp55E+e352ε33ε0cp66D=cp66E+e362ε33ε0cp56D=cp56E+e35e36ε33ε0h35=e35ε33ε0h36=e36ε33ε0β33S=1ε33ε0

The tangential components of the piezoelectric stress tensor:

(172) Tp5=cp55DS5+cp56DS6−h35D3=cp55Dy∂α∂x−cp56Dz∂α∂x−h35D3Tp6=cp56DS5+cp66DS6−h36D3=cp56Dy∂α∂x−cp66Dz∂α∂x−h36D3

The torque is:

(173) M=Mp+Mm=∫−b2b2dy∫z0−tpz0(yTp5−zTp6)dz+∫−b2b2dy∫z0z0+tm(yTm5−zTm6)dz==∫−b2b2dy∫z0−tpz0(y(cp55Dy∂α∂x+cp56Dz∂α∂x−h35D3)−z(cp56Dy∂α∂x+cp66Dz∂α∂x−h36D3))dz++∫−b2b2dx∫z0z0+tm(yGmy∂α∂x−z(−Gmz∂α∂x−q¯16h1))dz=Q˜∂α∂x+btp2〈h36〉D3+btm2〈q16〉h1

(174) Q˜=Qp+Qm,

where *z*_0_ is the position of the interface between the piezoelectric and magnetostrictive phases relative to the axis of rotation of the composite beam.

(175) 〈h36〉=1pt2∫z0−tp2−tp1z0zh36dz=(∫z0−tp2−tp1z0−tp1zhp236dz+∫z0−tp1z0zhp136dz)==1pt22(hp236tp2(2z0−2tp1−tp2)+hp136tp1(2z0−tp1))=14hp136

(176) 〈q16〉=1tm2∫z0z0+tmzq¯16dz=q¯16(2z0+tm)2tm,

where polar moments of the coefficient of shear stiffness of the piezoelectric and ferromagnet phases are given by:

(177) Qp=13cp66D(z03−(z0−tp)3)b+112cp55Dtpb3

(178) Qm=112Gmtmb3+13Gmb[(z0+tm)3−z03].

The voltage across the piezoelectric phase:

(179) U=∫z0−tpz0Ep3dz=∫z0−tpz0(−h35y∂α∂x+h36z∂α∂x+β33SD3)dz=tp2〈h36〉∂α∂x+tpβ33SD3.

The electrical displacement in the piezoelectric phase is given by the following equation:

(180) D3=Utpβ33S−tp〈h36〉β33S∂α∂x

Substituting in Equation (173), we find:

(181) M=Q∂α∂x+btp〈h36〉β33SU+btm2〈q16〉h1

(182) Q=Q˜−btp3〈h36〉2β33S.

The position of the interface between the piezoelectric and magnetostrictive phases relative to the axis of rotation of the composite beam *z*_0_ is determined from the condition of minimum effective shear modulus of the sample *Q*:

(183) ∂Q∂z0=0z0=cp66Dpt2−cm44mt22(cm44mt+cp66Dpt)

The equation of torsional vibrations is:

(184) $$J\frac{\partial \alpha^{2}}{\partial \tau^{2}}=\frac{\partial M}{\partial x},$$

where the moment of inertia of the sample per unit width:

(185) J=ρpIp+ρmIm,

where the polar moments of the piezoelectric and ferromagnetic are given by:

(186) Ip=13(z03−(z0−tp)3)b+112tpb3

(187) Im=13((z0+tm)3−z03)b+112tmb3

(188) $$J\frac{\partial \alpha^{2}}{\partial \tau^{2}}=Q\frac{\partial^{2}\alpha }{\partial x^{2}}.$$

The dependence of the twist angle on time is harmonic $\alpha ∼e^{i\omega t}$, therefore, we get:

(189) $$Q\frac{\partial^{2}\alpha }{\partial x^{2}}+J\omega^{2}\alpha =0,$$

(190) $$\frac{\partial^{2}\alpha }{\partial x^{2}}+k^{2}\alpha =0,$$

where the wave number is:

(191) $$k=\omega \sqrt{\frac{J}{Q}}.$$

The general solution of the equation of motion is:

(192) α=Acos(kx)+Bsin(kx),

*A*, *B* are unknown constants.

Boundary conditions for this case:

(193) $$\begin{array}{l} M\left(\frac{l}{2}\right)=0\\ M\left(-\frac{l}{2}\right)=0 \end{array}$$

(194) $$\frac{\partial \alpha }{\partial x}=k\left(B\cos\left(kx\right)-A\sin\left(kx\right)\right).$$

Then, we integrate Equation (179) over *x*:

(195) Ul=tp2〈h36〉α|−l2l2=2tp2〈h36〉Bsin(kl2).

Combining boundary conditions with Equation (195), we obtain a linear system of three inhomogeneous algebraic equations with three unknowns, *A*, *B*, *U*:

(196) Qk(Bcos(kl2)−Asin(kl2))+btp〈h36〉β33SU+btm2〈q16〉h1=0Qk(Bcos(kl2)+Asin(kl2))+btp〈h36〉β33SU+btm2〈q16〉h1=0Ul=2tp2〈h36〉Bsin(kl2)

Solving this system, the voltage across the piezoelectric can be obtained in the form:

(197) U=−2〈h36〉β33Stp2tm2b〈q16〉tan(kl2)Qklβ33S+2btp3〈h36〉2tan(kl2)h1.

The average electric field strength in the composite is:

(198) E¯3=Utm+tp=−2〈h36〉β33Stp2tm2b〈q16〉tan(kl2)(tm+tp)(Qklβ33S+2btp3〈h36〉2tan(kl2))h1.

As a result, the ME voltage coefficient is obtained as:

(199) αE=E¯3h1=−2〈h36〉β33Stp2tm2b〈q16〉tan(kl2)(tm+tp)(Qklβ33S+2btp3〈h36〉2tan(kl2)).

**Figure 18:**
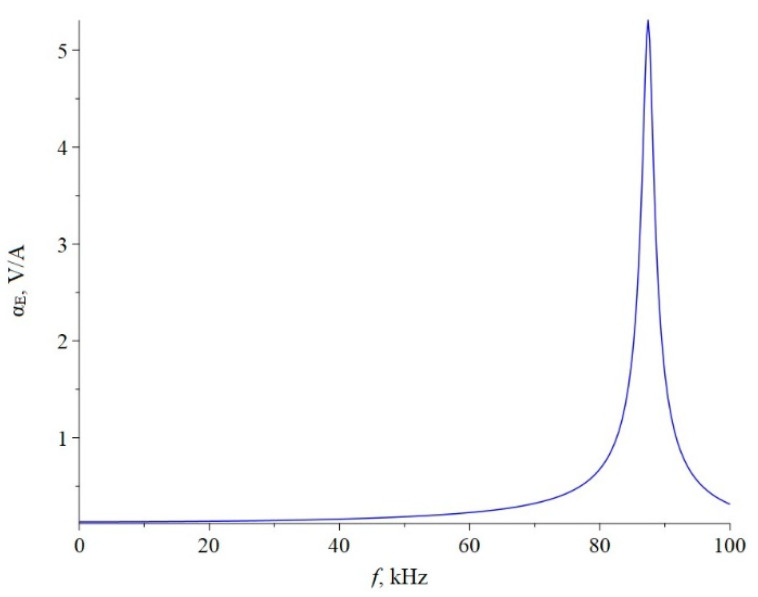
Theoretical dependence of the ME voltage coefficient on the frequency of the alternating magnetic field for ME structure Metglas/LiNbO_3_ Zyl + 45° in case of torsional mode.

Below, Figure 18 shows the dependence of the ME voltage coefficient on the frequency of the alternating magnetic field for case, when bimorph LiNbO_3_ Zyl + 45° are taken as the piezoelectric phase. Metglas is taken as the magnetostrictive phase. The length of ME composite was *l* = 23 mm, and width was *b* = 0.5 mm. In the calculation, the following material parameters of the initial components were used: for Metglas: *^m^ρ* = 7180 kg/m^3^, *^m^G* = 3.85·10^10^ Pa, *q*_16_ = 1.0·10^−9^ m/A, *^m^t* = 29 µm; for LiNbO_3_ Zyl + 45°: *^p^ρ* = 4647 kg/m^3^, *^p^c^E^*_55_ = 6.75·10^10^ Pa, *^p^c^E^*_56_ = 6.75·10^10^ Pa, *^p^c^E^*_55_ = 7.5·10^9^ Pa, *ε*_33_ = 36.5, *^p1^e*_35__=_ −^*p21*^*e*_35_ = 2.5 C/m^2^, *^p1^e*_36__=_ −^*p21*^*e*_36_ = 2.5 C/m^2^, *^p^t* = 0.4 mm. To take into account losses in the calculation, it is assumed: *ω* = 2*π*(1 + (1/2*Q*_*r*_)*i*)*f*, where *Q_r_* is the quality factor of the resonant system. The value of the quality factor was taken the same as for the longitudinal mode *Q_r_* = 100.

The fundamental resonant frequency for this case is:

(200) $$f_{r}=\frac{1}{2l}\sqrt{\frac{Q}{J}}.$$

#### 3.3.2. Quasi-Static Mode

In a magnetostrictive-piezoelectric structure based on bimorph lithium niobate, the longitudinal-shear mode is not excited due to oppositely directed polarization in the layers of lithium niobate. Therefore, the expression for the ME voltage coefficient in the quasi-static mode can be obtained from Equation (199) by assuming the frequency f equal to zero:

(201) αE=−〈h36〉β33Stp2tm2b〈q16〉(tm+tp)(Qβ33S+btp3〈h36〉2).

**Figure 19:**
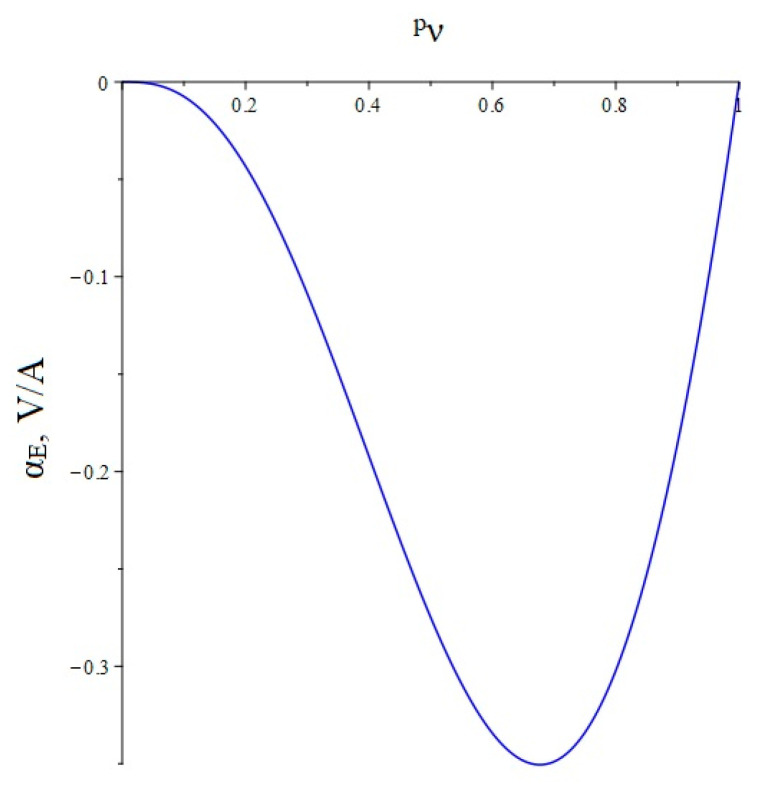
Theoretical dependence of the ME voltage coefficient on the volume fraction of the piezoelectric material for the asymmetric ME structure Metglas/LiNbO_3_ Zyl + 45° for the quasi-static torsional mode.

Below, Figure 19 shows the dependence of the ME voltage coefficient on the volume fraction of the piezoelectric material for the asymmetric ME structure Metglas/LiNbO_3_ Zyl + 45° for the quasi-static torsional mode.

## 4. Discussion

It is necessary to discuss the accuracy of the above formulas for the fundamental resonant frequencies for various modes. In the case of a longitudinal mode, we turn to the expression for the ME voltage coefficient Equation (35). Obviously, the resonant frequency should vanish from the denominator of this expression. However, this denominator consists of the main term and term proportional to the dimensionless quantity of $d_{31}^{2}/\left(\varepsilon \varepsilon_{0}{}^{p}S_{11}\right)$. If this dimensionless quantity is small compared to unity, then the corresponding term in the denominator can be neglected, and then Equation (36) is obtained for the fundamental resonant frequency for the longitudinal mode.

If we estimate the value of the dimensionless quantity of $d_{31}^{2}/\left(\varepsilon \varepsilon_{0}{}^{p}S_{11}\right)$, then for PZT we get 0.13, and for LN y + 128° 0.24. In this case, the main resonant frequency, determined from the exact plot for PZT Figure 2, is 158 kHz, determined according to the approximate Equation 36 of 151 kHz. Additionally, the main resonant frequency, determined from the exact graph for LN y + 128° Figure 2, is of 296 kHz, and determined from the approximate Equation 36, is 267 kHz. Obviously, the larger the value of the dimensionless quantity of $d_{31}^{2}/\left(\varepsilon \varepsilon_{0}{}^{p}S_{11}\right)$, the more the exact fundamental resonant frequency differs from the fundamental resonant frequency. To discuss this issue for the bending mode in the case of a sample with free ends, we turn to the expression for the ME stress coefficient Equation (65). Likewise, the resonant frequency must vanish from the denominator of this expression. Similarly, the denominator consists of a principal term and a term proportional to the dimensionless quantity of ${}^{p}t_{3}{\langle h_{31}\rangle }^{2}/\left(t^{3}\langle c_{11}\rangle \beta_{33}^{S}\right)$. If one estimates the value of the dimensionless quantity of ${}^{p}t_{3}{\langle h_{31}\rangle }^{2}/\left(t^{3}\langle c_{11}\rangle \beta_{33}^{S}\right)$, then for PZT it will be 0.0023, and for LN y + 128° it will be 0.0011. In this case, the main resonant frequency, determined from the exact graph for PZT Figure 6, is of 17,512 Hz, and determined from the approximate Equation (66) is of 17,498 Hz. Additionally, the main resonant frequency, determined from the exact graph for LN y + 128° Figure 6, is of 33,098 Hz, and determined from the approximate Equation (66) of 33,086 Hz. Since the values of the dimensionless quantity are much less than unity for PZT and LN y + 128°, the differences between the exact fundamental resonant frequencies and those determined by the approximate formula are negligible.

For the case of a bending mode for a sample with an ME cantilever, the voltage coefficient is determined by Equation (72), and the approximate formula for the fundamental resonant frequency is Equation (73). Obviously, the results for this case will be completely similar to the results for the case for the bending mode for a sample with free ends. To discuss this issue for the torsional mode, we turn to the expression for the ME voltage coefficient Equation (151). Likewise, the resonant frequency must vanish from the denominator of this expression. Similarly, the denominator consists of a principal term and a term, proportional to the dimensionless quantity of ${}^{p}t_{3}{\langle h_{31}\rangle }^{2}/\left(t^{3}\langle c_{11}\rangle \beta_{33}^{S}\right)$. The value of this dimensionless coefficient is much less than unity. Therefore, the main resonant frequency, determined by the exact graph Figure 15, of 67,809.0 Hz, and determined by the approximate Equation (152) of 67,809.7 Hz, practically do not differ.

Similarly, questions of accuracy are considered for approximate Equations (115), (152), and (200) for the main resonant frequencies.

In addition, the correspondence of the described theory to the experimental data should be noted. The longitudinal and bending modes of the ME effect have been fairly well studied experimentally. The obtained experimental results are in good agreement with the stated theory [12,13]. The experimental study of the torsional mode of the ME effect is just beginning, so it is not yet possible to draw reasonable conclusions about the correspondence of the theory presented to the experimental data.

## 5. Conclusions

The article considers the theory of low-frequency direct ME effect in symmetric and asymmetric magnetostrictive-piezoelectric structures in longitudinal and bending, as well as longitudinal-shear and torsional modes. Expressions are obtained for the ME voltage coefficients in the quasi-static and EMR modes. Additionally, for the EMR mode, approximate formulas for the main resonant frequencies were obtained and their accuracy was investigated. For the torsion mode, the advantages of using a bimorph piezoelectric material are shown, which led to a significant increase in the ME voltage coefficient. A comparison of the obtained theoretical results with known data from the literature and experiment for the GaAs-Metglas and LiNbO_3_-Metglas structures showed satisfactory agreement. The value of the study, according to the authors, lies in the fact that within the framework of a unified approach, the main relationships for the ME voltage coefficients for all modes of low-frequency direct ME effect were obtained. The results obtained can be used for choosing ME composites that can create new ME devices in the low-frequency range. In terms of further research, it is of interest to carry out a similar calculation of the inverse low-frequency ME effect and compare the results obtained.

## Data Availability

Not applicable.
